# Supplementary material for: Top-Down Inhibition of BMP Signaling Enables Robust Induction of hPSCs Into Neural Crest in Fully Defined, Xeno-free Conditions
Source: Stem Cell Reports. 2017 Sep 14;9(4):1043–52. doi: 10.1016/j.stemcr.2017.08.008 (PMC5639211; doi:10.1016/j.stemcr.2017.08.008)
Supplement: Document S1. Supplemental Experimental Procedures and Figures S1–S4 [file mmc1.pdf]

Stem Cell Reports, Volume *9*

Supplemental Information

Top-Down Inhibition of BMP Signaling Enables Robust Induction of hPSCs Into Neural Crest in Fully Defined, Xeno-free Conditions

James O.S. Hackland, Tom J.R. Frith, Oliver Thompson, Ana Marin Navarro, Martin I. Garcia-Castro, Christian Unger, and Peter W. Andrews

Supplemental Data

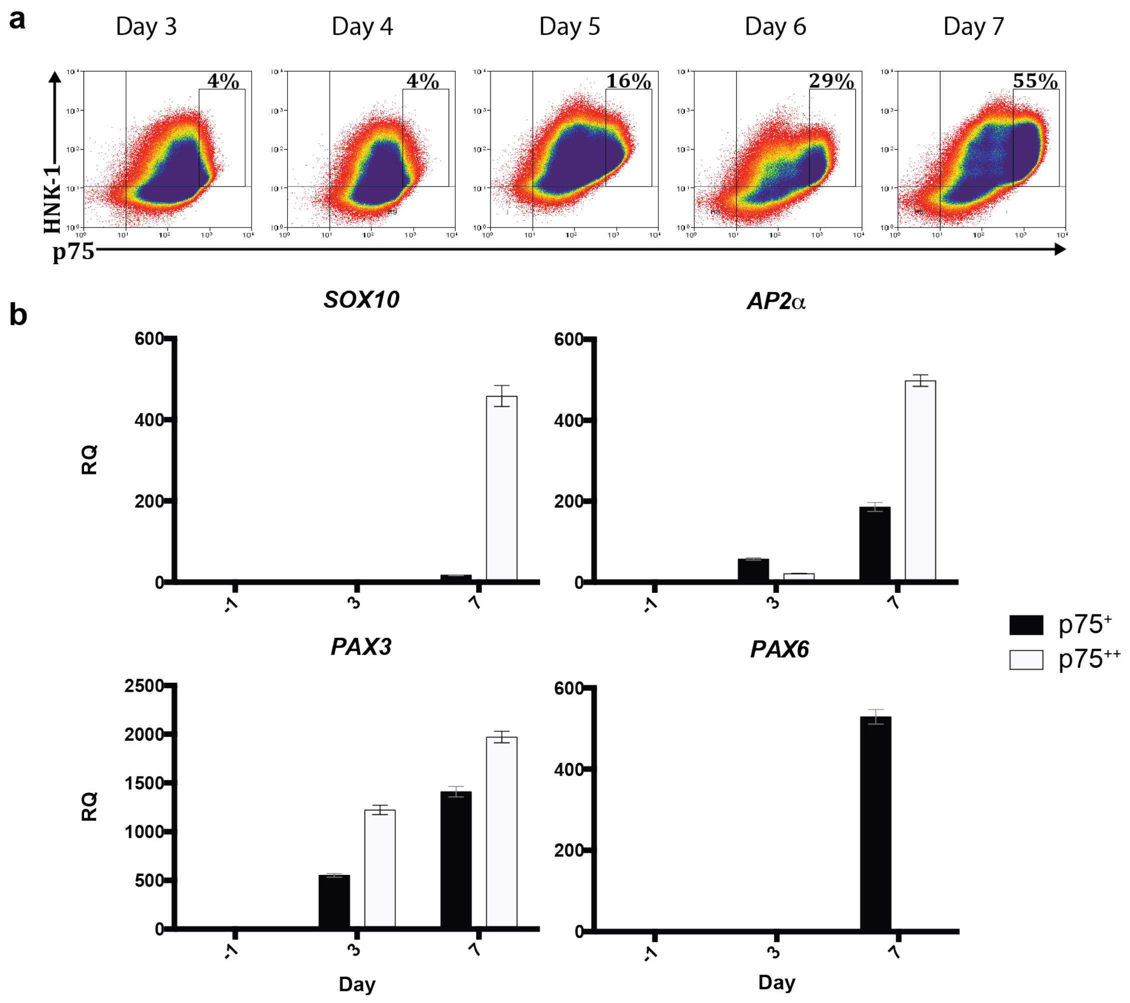

Figure S1. Gene expression in p75 high/low (p75^{++}/p75^{+}) during neural crest induction of Shef6 using NCN2. Related to Figure 1. (**a**) The surface marker p75 is upregulated during neural crest induction and (**b**) can be used to purify the culture for *SOX10* expressing cells in the absence of a *SOX10* reporter construct. Data represents a single experiment showing typical distribution of gene expression between populations.

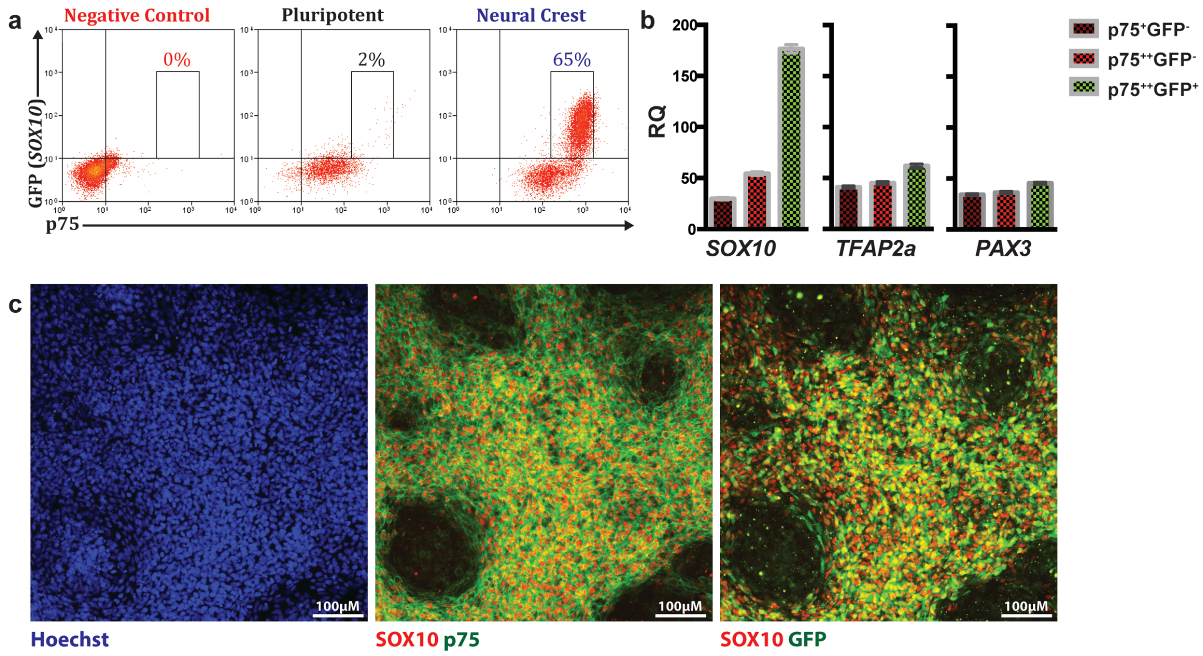

Figure S2. Expression of surface markers and neural crest-associated genes in H9:*SOX10*. Related to Figures 2, 3 & 4. (a) Expression of GFP and p75 in pluripotent cells and after 7 days of neural crest differentiation. (b) Expression of neural crest-associated genes after FACS in p75+, p75++ and p75++GFP+ populations after neural crest differentiation. Data represents a single experiment showing technical replicates. (c) Expression of SOX10, p75 and GFP in a typical culture at day 7 of neural crest differentiation.

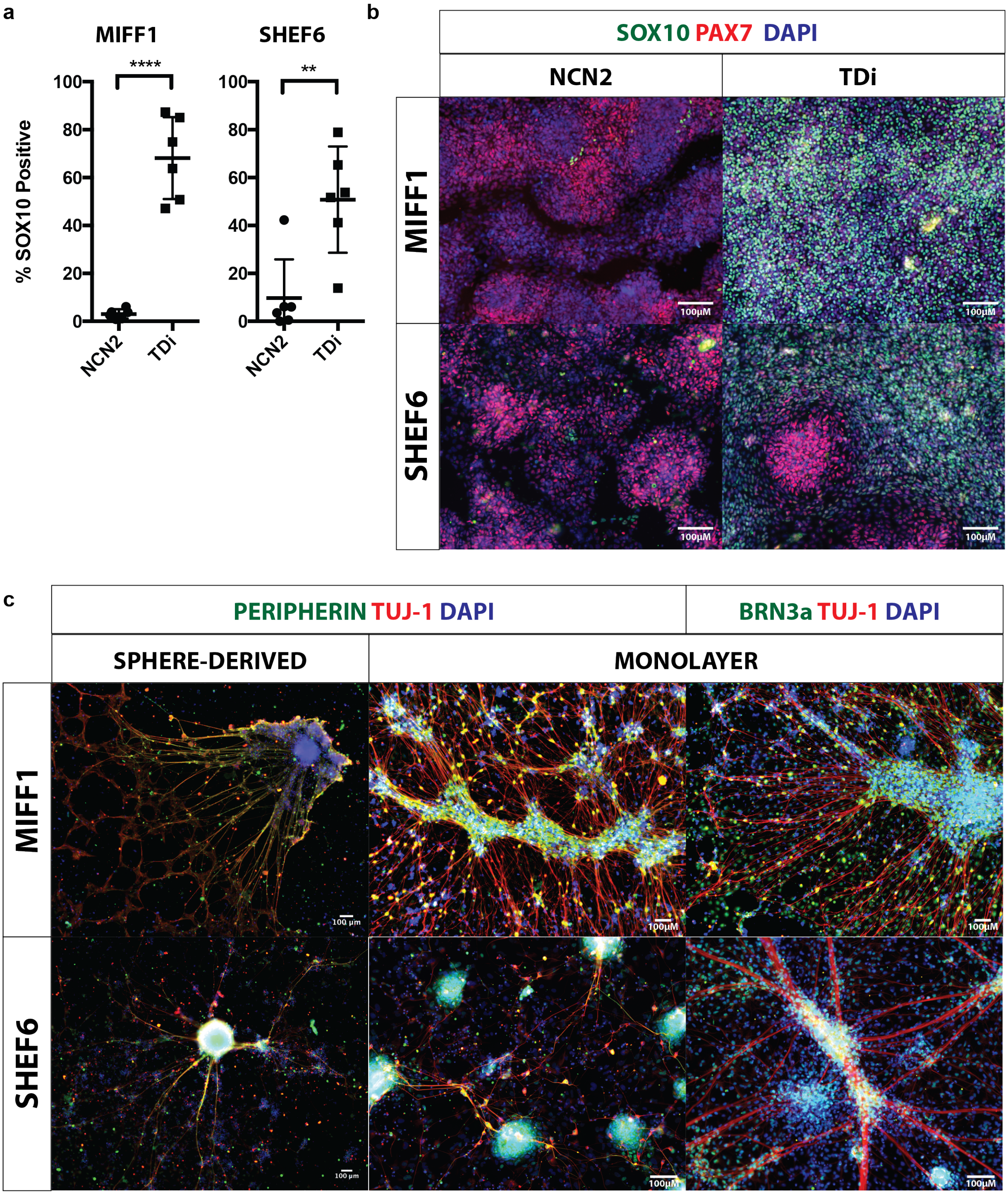

Figure S3. Neural crest induction using TDi in Shef6 (hESC) and Miff-1 (hiPSC). Related to Figure 4. (**a**) Use of TDi during neural crest induction significantly improves the reproducibility of differentiation in both Shef6 and Miff-1. Each point represents a single experiment. Error bars represent SD. $^{*}P \leq 0.05$, $^{**}P \leq 0.01$, $^{***}P \leq 0.001$, $^{****}P \leq 0.0001$. Efficiency of induction was calculated by applying Nikon Elements image analysis software to immunofluorescence data examples of which are shown in (**b**). (**b**) Typical expression of neural crest markers SOX10 and PAX7 after 7 days of induction using either NCN2 or TDi. (**c**) Neural crest generated from Shef6 and Miff-1 respond to neural induction conditions, either in monolayer or from sphere culture, by expressing the peripheral neural marker Peripherin and the sensory neural marker Brn3a.

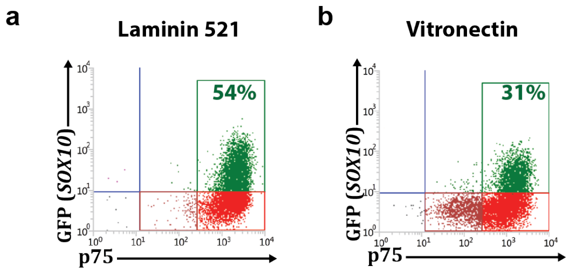

Figure S4. Neural Crest differentiation on other matrices. Related to differentiation in fully defined and xeno-free conditions. Neural crest differentiation of H9:*SOX10* on fully defined and xeno-free matrices Laminin-521 and vitronectin using TDi.

Antibody	Supplier/Catalog Number
**Ms anti PAX3**	**DSHB/AB528426**
**Ms anti PAX6**	**Santa Cruz/sc81649**
**Rb anti SOX10**	**Cell signaling/89356S**
**Rb anti SOX2**	**Cell signaling/3579P**
**Rb anti p75**	**Cell signaling/8238**
**Ms anti HNK-1**	**Sigma/C6680**
**Chk anti GFP**	**Abcam/ab13970**
**Ms anti S100b**	**Sigma/S2532**

List of antibodies used for FACS and immunofluorescence.

Gene	Assay ID
SOX10	**Hs00366918_m1**
TFAP2a	**Hs01029413_m1**
PAX3	**Hs00240950_m1**
PAX6	**Hs01088114_m1**
SOX2	**Hs01053049_s1**
GATA2	**Hs00231119_m1**
GATA3	**Hs00231122_m1**
OCT4 (POU5F1)	**Hs04260367_gH**

List of Taqman Gene Expression Assay mixes used (Catalog Number 4331182).

Supplemental Experimental Procedures

Pluripotent Stem Cell Culture

WA09 (H9) (Thomson et al. 1998), Shef6 (Aflatoonian et al. 2010), and H9:*SOX10* (Chambers et al. 2012) hESC, and Miff-1 (Desmarais et al. 2016), NB1 (unpublished, fully characterized) hiPSC, were cultured in mTESR on matrigel. All cells used had a normal diploid karyotype.

Neural Crest Differentiation

N2 supplement was sourced from ThermoFisher (17502048), CHIR99021, SB431542 and DMH1 were sourced from Tocris (4425, 1614, 4126 respectively) and BMP4 was from Life Technologies (PHC9534).

Immunofluorescence

Cultures were fixed in 4% paraformaldehyde for 10 minutes before permeabilization and blocking for 1 hour (1% BSA, 10% goat serum, 0.3M glycine and 0.1% Tween-20 in PBS). Primary and secondary antibody incubation was carried out in the same buffer overnight and for 1 hour respectively (see supplemental table). Hoechst 33342 (H3570 - Life Technologies) was used as a DNA stain. Images were taken using an InCell Analyser 2200 (GE Healthcare Life Sciences). For the data presented in Supplemental Figure 3 images were taken using a Nikon Eclipse Ti and analyzed using Nikon Elements image analysis software. These data were calculated as follows: Each biological replicate shown represents the mean expression values of three technical replicates each of which represent four preset locations within each well. The positive/negative intensity threshold was set in each case with the aid of an additional well without primary antibody.

Flow Cytofluorimetry

Flow cytometry analysis was carried out as previously described (Draper et al. 2002). Briefly, cultures were treated with Accutase (Gibco - A11105-01) and both primary and secondary antibody incubation was carried out in in FACS buffer (10% FCS, PBS) for 15 minutes (see supplemental table). Cells were analyzed using a BD FACSJazz (BD Biosciences). For negative controls the antibody produced by the parent myeloma P3X63AG8 was used (Kohler & Milstein 1975; Draper et al. 2002).

qPCR

RNA was extracted from whole cultures or sorted populations using TRIzol (15596018 - Life Technologies) and cDNA was generated using Multiscribe reverse transcriptase (4311235 - Life Technologies). qPCR was carried out using 4ng

of cDNA per 10ml reaction with Taqman Fast Universal PCR master mix (1411408 - Life Technologies) and pre-optimized Gene Expression Assay mixes (4331182 - Life Technologies; supplementary table) before analysis using the QuantStudio 12K Flex Real-Time PCR system (4471087 - Life Technologies). Data are presented either as difference in cycle threshold between the gene of interest (GOI) and β-Actin (ΔcT) or a relative quantification (RQ) of the change in gene expression between the initial pluripotent culture and the differentiated culture (RQ; 2^-ΔΔcT).

Terminal Differentiation of Neural Crest

Terminal differentiation was performed on GFP^+ sorted putative neural crest derived from H9:*SOX10* unless otherwise stated. For mesectoderm differentiation, cells were plated out in MesenCult™-ACF (StemCell Technologies Cat# 05449). After 10 days, medium was changed to MesenCult™-ACF Chondrogenic Differentiation Medium (StemCell Technologies Cat#05455) or Osteogenic Stimulatory Kit (StemCell Technologies Cat# 05404) and analyzed using Toluidine Blue (Acris Bioscience) Alizarin Red staining (Sigma A5533).

Terminal differentiation into neural lineages was carried out as described in Lee (2010) either as a monolayer or as neural crest spheres directly from non-adherent culture. Plates were coated with Fibronectin (Sigma F1141), Laminin (Cultrex 3400-010-01) and Poly-L-Ornithine (Sigma P4957). FGF, EGF, GDNF, BDNF and NGF were sourced from peprotech and dbCAMP from Sigma (D0627). The BRN3a and ISL1 expression shown in Figure 2d and Supplemental Figure 3 was achieved using the modified version of this protocol described in (Chambers et al. 2012).
