# Supplementary material for: Top-Down Inhibition of BMP Signaling Enables Robust Induction of hPSCs Into Neural Crest in Fully Defined, Xeno-free Conditions
Source: Stem Cell Reports. 2017 Sep 14;9(4):1043–52. doi: 10.1016/j.stemcr.2017.08.008 (PMC5639211; doi:10.1016/j.stemcr.2017.08.008)
Supplement: Document S2. Article plus Supplemental Information [file mmc2.pdf]

Stem Cell Reports
Report

ISSCR

OPEN ACCESS

Top-Down Inhibition of BMP Signaling Enables Robust Induction of hPSCs Into Neural Crest in Fully Defined, Xeno-free Conditions

James O.S. Hackland,[1,2,*] Tom J.R. Frith,[2] Oliver Thompson,[2] Ana Marin Navarro,[3] Martin I. Garcia-Castro,[1] Christian Unger,[2] and Peter W. Andrews[2]
[1]University of California Riverside, Department of Biomedical Sciences, Riverside, CA 92521, USA
[2]University of Sheffield, Department of Biomedical Science, Sheffield S10 2TN, UK
[3]Karolinska Institutet, Department of Neuroscience, Stockholm, Sweden
*Correspondence: hacklandj@gmail.com
http://dx.doi.org/10.1016/j.stemcr.2017.08.008

SUMMARY

Defects in neural crest development have been implicated in many human disorders, but information about human neural crest formation mostly depends on extrapolation from model organisms. Human pluripotent stem cells (hPSCs) can be differentiated into *in vitro* counterparts of the neural crest, and some of the signals known to induce neural crest formation *in vivo* are required during this process. However, the protocols in current use tend to produce variable results, and there is no consensus as to the precise signals required for optimal neural crest differentiation. Using a fully defined culture system, we have now found that the efficient differentiation of hPSCs to neural crest depends on precise levels of BMP signaling, which are vulnerable to fluctuations in endogenous BMP production. We present a method that controls for this phenomenon and could be applied to other systems where endogenous signaling can also affect the outcome of differentiation protocols.

INTRODUCTION

Neural crest, a transient, migratory tissue arising during vertebrate development, is first observed in the human at Carnegie stage 9 (O'Rahilly and Müller, 2007). Defects in the development of the tissue can lead to neurocristopathies with a wide range of characteristics. For ethical reasons, the study of human neural crest induction, and human ectoderm development in general, has been largely restricted to observational work with human embryos (Betters et al., 2010; O'Rahilly and Müller, 2007) and to studies of *in vitro* differentiation of human pluripotent stem cells (hPSCs). The latter have been carried out to generate neural (Chambers et al., 2009), neural crest (Menendez et al., 2011; Mica et al., 2013; Leung et al., 2016), placodal (Chen et al., 2012), and non-neural ectoderm (Metallo et al., 2010) lineages. *In vivo*, human neural crest expresses many of the markers found in other vertebrates, including *SOX10*, *PAX3*, *TFAP2a*, and p75 (Betters et al., 2010), and when hPSCs are differentiated into neural crest they share this expression profile and can generate neural crest-derived lineages *in vitro* (Menendez et al., 2011; Mica et al., 2013). Systems such as these not only hold potential for developing an understanding of neural crest formation in humans but also for generating cells for the treatment of neurocristopathy.

In vertebrate ectoderm, BMP signaling proteins play a key role in medio-lateral patterning. Neural crest induction in avians and amphibians, which was originally thought to occur through a process of interaction between neurectoderm and the developing epidermis (Selleck and Bronner-Fraser, 1995), is now understood to occur during gastrulation in a manner dependent on BMP, as well as other signals (Basch et al., 2006). Antagonists produced by the dorsal mesoderm modulate BMP activity across the ectoderm differentially specifying neural, neural crest, placodal, and epidermal lineages (Hemmati-Brivanlou et al., 1994; Furthauer et al., 1999; Zimmerman et al., 1996; Sasai et al., 1996; Piccolo et al., 1996; Lamb et al., 1993). These antagonists generate the intermediate levels of BMP activity required during gastrulation for specifying neural crest fate in fish and amphibians (Tribulo et al., 2003; Wilson et al., 1997; Marchant et al., 1998; Barth et al., 1999; Nguyen et al., 1998; Faure et al., 2000). During gastrulation, neural crest features can be induced elsewhere in the ectoderm of amphibians by manipulating BMP levels in a context-dependent manner: In the epidermis, BMP inhibition induces *snai2* expression (Steventon et al., 2009), and the prospective neural plate domain shrinks after unilateral injection of BMP4 mRNA deflects the *snai2* expression domain medially (LaBonne and Bronner-Fraser, 1998). After this early stage of specification, BMP activity at the neural plate border increases (Faure et al., 2002; Nguyen et al., 1998; Steventon et al., 2009; Faure et al., 2000), and the ability of the ectoderm to respond to BMP changes; instead of repressing neural identity, BMP adopts a dorsoventral patterning role for the neural tube (Timmer et al., 2002).

Although it is clear that BMP activity affects the differentiation of hPSCs into neural crest, the dynamics of this process are still poorly understood. Specifically, the optimal level of BMP activity required for neural crest induction is

This is an open access article under the CC BY license (http://creativecommons.org/licenses/by/4.0/).

unknown. The reason for this is that BMP ligands can be present in media components or produced endogenously by the cells (Pera et al., 2004), and current methods of neural crest differentiation have multiple confounding factors that make it difficult to assess the BMP environment of the culture. These include the use of serum, non-recombinant BSA, or other animal-derived products, as well as passaging during the differentiation process. For these reasons, some approaches require the inhibition of BMP (Lee et al., 2010; Mica et al., 2013), whereas others have shown that BMP inhibition represses neural crest formation (Menendez et al., 2011; Leung et al., 2016). It has been proposed that these differences are due to a temporal effect necessitating BMP inhibition early during differentiation (Mica et al., 2013). However, BMP inhibition can repress neural crest fates even when applied for just the first 24 hr of hPSC differentiation, suggesting otherwise (Leung et al., 2016). The development of a system without these caveats is key to a better understanding of the differentiation process and a requirement for translation of these protocols to the clinic. A number of neurocristopathies are good candidates for cell replacement therapies, such as Hirschsprung's disease (Fattahi et al., 2016; Workman et al., 2016), for which a fully defined and xeno-free approach to generating the required cells is essential.

In this study, we describe a fully defined and xeno-free system for the differentiation of hPSCs into neural crest and show that an optimum level of BMP activity is required for neural crest induction, whereas higher or lower levels lead to the induction of genes associated with non-neural or neural identity, respectively. Finally, we show that variations in endogenous BMP production adversely affect neural crest differentiation efficiency between iterations of the same protocol, and we present a method that controls for this effect in multiple hPSC lines.

RESULTS

To establish a fully defined protocol for neural crest differentiation, we modified the dual SMAD inhibition and WNT activation approaches described previously (Lee et al., 2010; Menendez et al., 2011) and developed an induction medium (NCN2) that utilizes inhibition of TGFβ and GSK3 without the inhibition of BMP signaling, as this was found to repress neural crest differentiation. To initiate differentiation, human embryonic stem cells (hESCs; WA09 or Shef6) or human induced pluripotent stem cells (hiPSCs; Miff-1) were seeded at 10,000 cells cm^{-2} on Matrigel in E6 medium supplemented with 10 μM γ-27632. After 24 hr, the culture was switched to NCN2 medium containing N2 supplement, CHIR99021 (1.0 μM), and SB431542 (2.0 μM). In each cell line, sustained culture in NCN2 for 7 days resulted in expression of the surface markers p75 and HNK-1 and the transcription factors *TFAP2a*, *PAX3*, and *SOX10*, all of which are associated with neural crest identity. In addition, expression of the stem cell marker *OCT4* (*POU5F1*) was lost (Figures 1A–1C). Sorting for cells with high p75 expression ($p75^{++}$) isolated most of the *SOX10* expression within the culture, confirming that this surface marker can be used to purify putative neural crest as has been shown before (Liu et al., 2012; Figure S1). To confirm neural crest identity, we further tested differentiation of cells sorted on the basis of GFP expression from a *SOX10* reporter cell line (H9:*SOX10;* Figure S2) into neural crest-derived cell types. These included mesenchymal cells that express CD73, CD90, and CD105 (Figure 2A) and differentiate into osteocytes and chondrocytes (Figure 2B). In addition, day 7 putative neural crest could be differentiated into peripheral neurons expressing β-tubulin and peripherin (Figure 2C), as well as sensory neurons positive for BRN3a and ISL1 (Figure 2D) and putative glial cells expressing S100b and SOX10 (Figure 2E). Day 7 putative neural crest also expressed the cranial marker ETS-1 (Figure 2F) and displayed a high level of motility *in vitro* (Figure 2G). Differentiation into melanocytes using the protocol described in Mica et al. (2013) was also attempted but MITF expression was not observed.

Prolonged culture of hPSC-derived neural crest was possible by detaching the cells at day 7 and culturing in non-adherent conditions. In this environment, the cells formed spheres that could be grown indefinitely. SOX10 expression was retained for at least 4 weeks while expression of PAX3 was lost, possibly indicating a reduction in the proportion of neural crest precursors (Figure 2H). When plated onto Matrigel, these spheres attach and cells migrate out as if from an explanted neural tube (data not shown).

The functional characteristics and marker expression of cells generated using NCN2 are analogous to that of neural crest, but the system is unpredictable. Cultures exhibited a high level of variability in differentiation efficiency ($p75^{++}$ cells ranging from 5% to 80%) in ostensibly identical conditions. Instances of low differentiation efficiency often coincided with increased expression of transcription factors associated with other ectodermal tissues, such as the neural markers *SOX2* and *PAX6* or the non-neural markers *GATA2* and *GATA3*, at the expense of the neural crest marker *SOX10*. Since BMP signaling plays a role in the patterning of early ectoderm, while BMP inhibition has a repressive effect in our system, we postulated that variation in endogenous BMP signaling might be the cause of the variability in differentiation efficiency. To investigate this, we used recombinant BMP4 and noggin to modulate BMP signaling during differentiation and analyzed cell identity based on expression of ectodermal

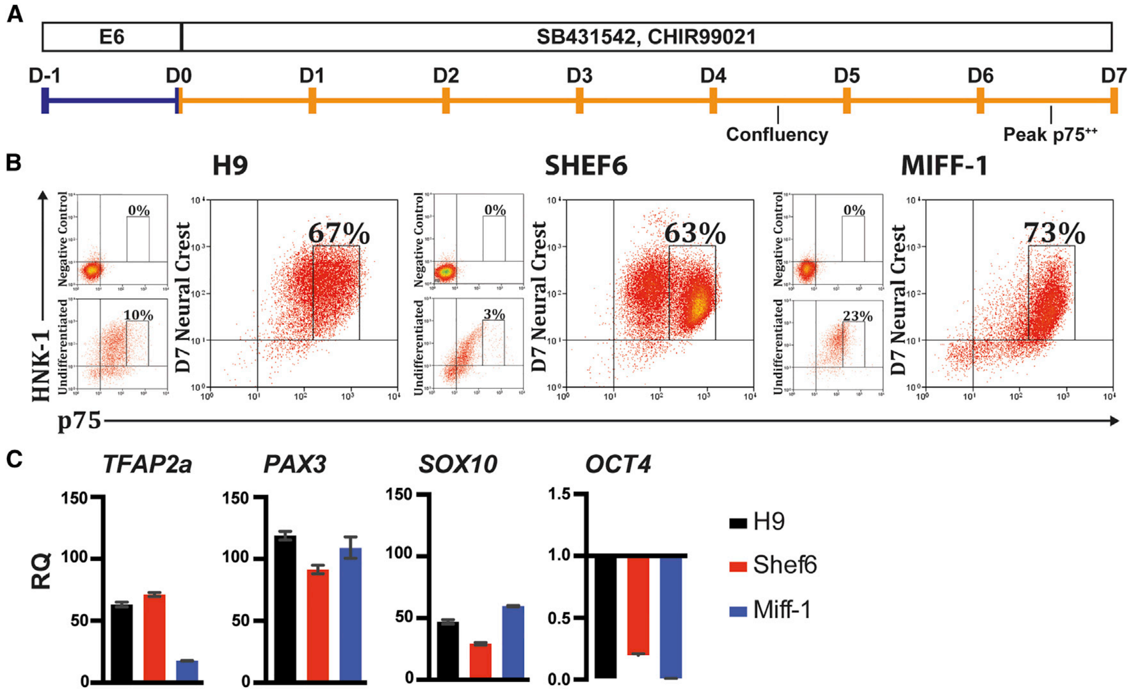

Figure 1. Differentiation of hPSCs into Neural Crest

(A) Schematic of the neural crest differentiation protocol. Blue, E6 medium; orange, NCN2 induction medium.

(B) hESC Shef6 and H9 (WA09) and hiPSC Miff-1 upregulate p75 after 7 days in NCN2; expression is low in undifferentiated cells.

(C) Expression of *TFAP2a*, *PAX3*, and *SOX10* at day 7 (qPCR). The stem cell marker OCT4 is repressed. Gene expression is presented relative to initial undifferentiated hPSCs (RQ). Error bars represent SD. Data for each cell line is from a single experiment representing the potential differentiation efficiency obtainable. Pooled data were not used because of reproducibility issues when using NCN2 alone as described and addressed below.

markers: *SOX2*, *PAX6*, and *PAX3* for neural; *PAX3, SOX10*, and *TFAP2a* for neural crest; and *TFAP2a*, *GATA2*, and *GATA3* for non-neural. In addition, we used the reporter line H9:*SOX10* to provide a better assessment of differentiation efficiency, as p75 is expressed in neural tissue as well as neural crest.

A series of experiments were conducted in which parallel cultures were subjected to different levels of BMP signaling throughout differentiation. These experiments were assessed retrospectively to determine if (1) instances of low neural crest differentiation efficiency in NCN2 correlated with low/high endogenous BMP expression levels and could be rescued by exogenous BMP modulation in parallel cultures and (2) repeat iterations of differentiation showed a requirement for different levels of BMP4 or noggin for efficient neural crest differentiation. These experiments revealed that peak differentiation efficiency, as assessed by p75^{++} and GFP expression, could occur under different exogenous BMP conditions (i.e., different levels of recombinant BMP4 or noggin in the differentiation medium) between experiments. Data are presented from two examples that exhibited different differentiation efficiencies when in NCN2 medium alone, 24% and 4% GFP positive for experiment 1 and 2, respectively (Figure 3A). In experiment 1, a significantly higher level of *BMP4* expression was detected from day 0 to 4 of differentiation than in experiment 2 (Figure 3B). This disparity between the two experiments correlated with higher levels of endogenous BMP4 protein detected in the medium by ELISA on day 0 in experiment 1 (Figure 3C). In experiment 2, a parallel culture grown in NCN2 with additional 1 ng mL^{-1} BMP4 exhibited differentiation efficiency equivalent to that of NCN2 alone in experiment 1. In contrast to this, additional 1 ng mL^{-1} BMP4 in experiment 1 reduced efficiency (Figure 3A). In both cases, the efficiency in the presence of 2ng mL^{-1} BMP4 was 0%, and the effect of noggin alone was repressive. This is consistent with insufficient BMP activity in the basal condition of experiment 2, compared with experiment 1. This allows the addition of recombinant BMP4 in experiment 2 to increase the BMP activity into the range required for neural crest, equivalent to the basal condition in experiment 1, resulting in neural crest induction. In either case, further addition of BMP4 appears to result in a level of BMP signaling that is too high for neural crest induction.

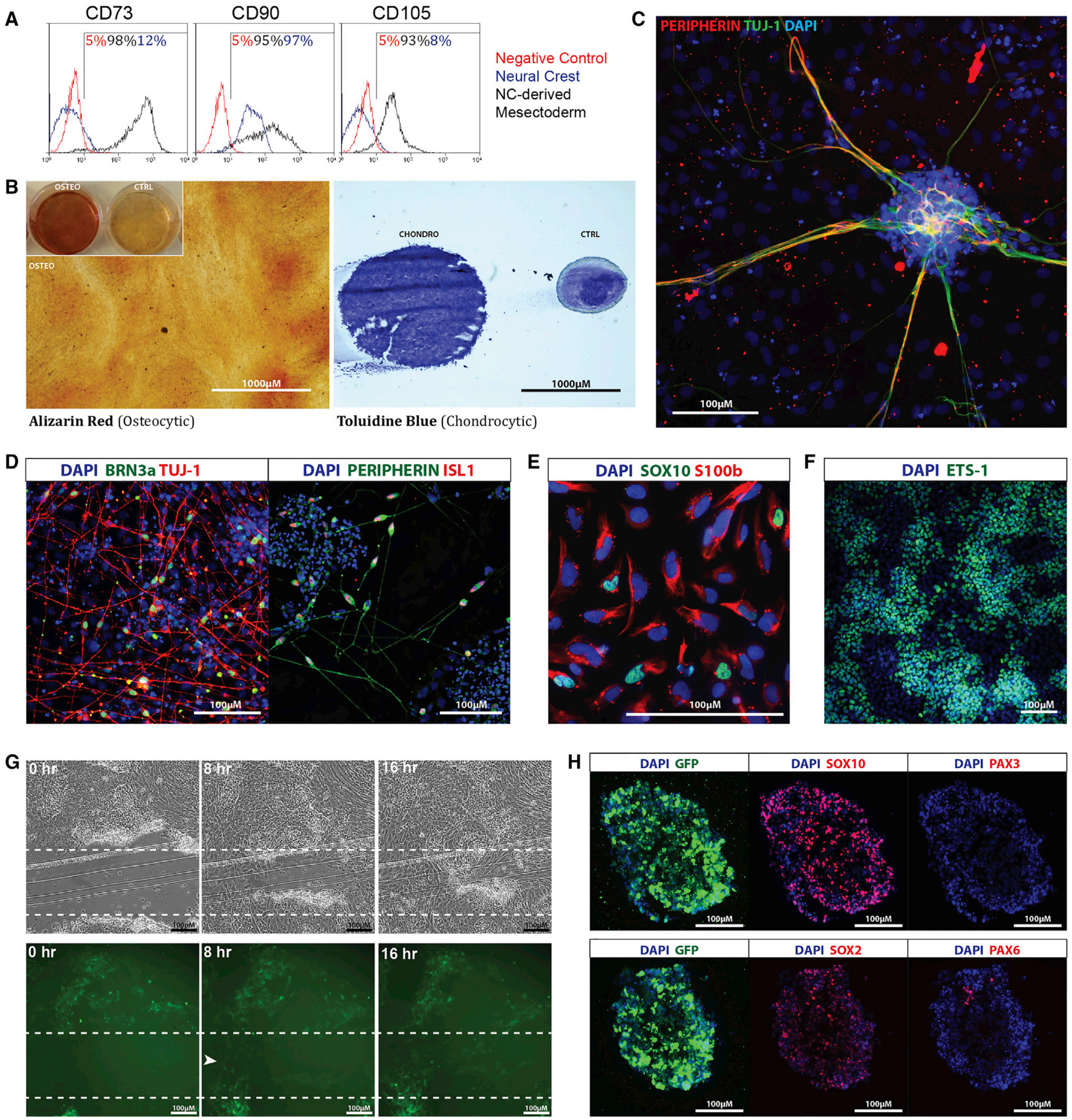

Figure 2. Analysis of hPSC-Derived Neural Crest

(A and B) Putative neural crest derived from H9:*SOX10* and sorted for GFP expression (A) upregulates CD73, CD90, and CD105 in mesenchymal growth conditions (see Experimental Procedures) and (B) can be differentiated into osteocytes and chondrocytes, stained with Alizarin red and toluidine blue, respectively.

(C and D) H9:*SOX10*-derived neural crest in neural differentiation conditions are positive for peripherin and TUJ-1 in unsorted cells via intermediate spheroid culture (C) and in sorted cells alongside sensory neural markers BRN3a and ISL1 (D).

(E) Expression of the glial markers SOX10 and S100b in neural crest derived from the iPSC line NB1.

(F) Expression of the cranial neural crest marker ETS-1 at day 7 of neural crest induction (Miff-1).

(G) *In vitro*, putative neural crest are highly motile; scratch ablation recolonized by GFP positive cells (arrow) within 16 hr (H9:*SOX10*).

(H) Sections of typical H9:*SOX10*-derived neural crest spheres after 4 weeks of non-adherent culture.

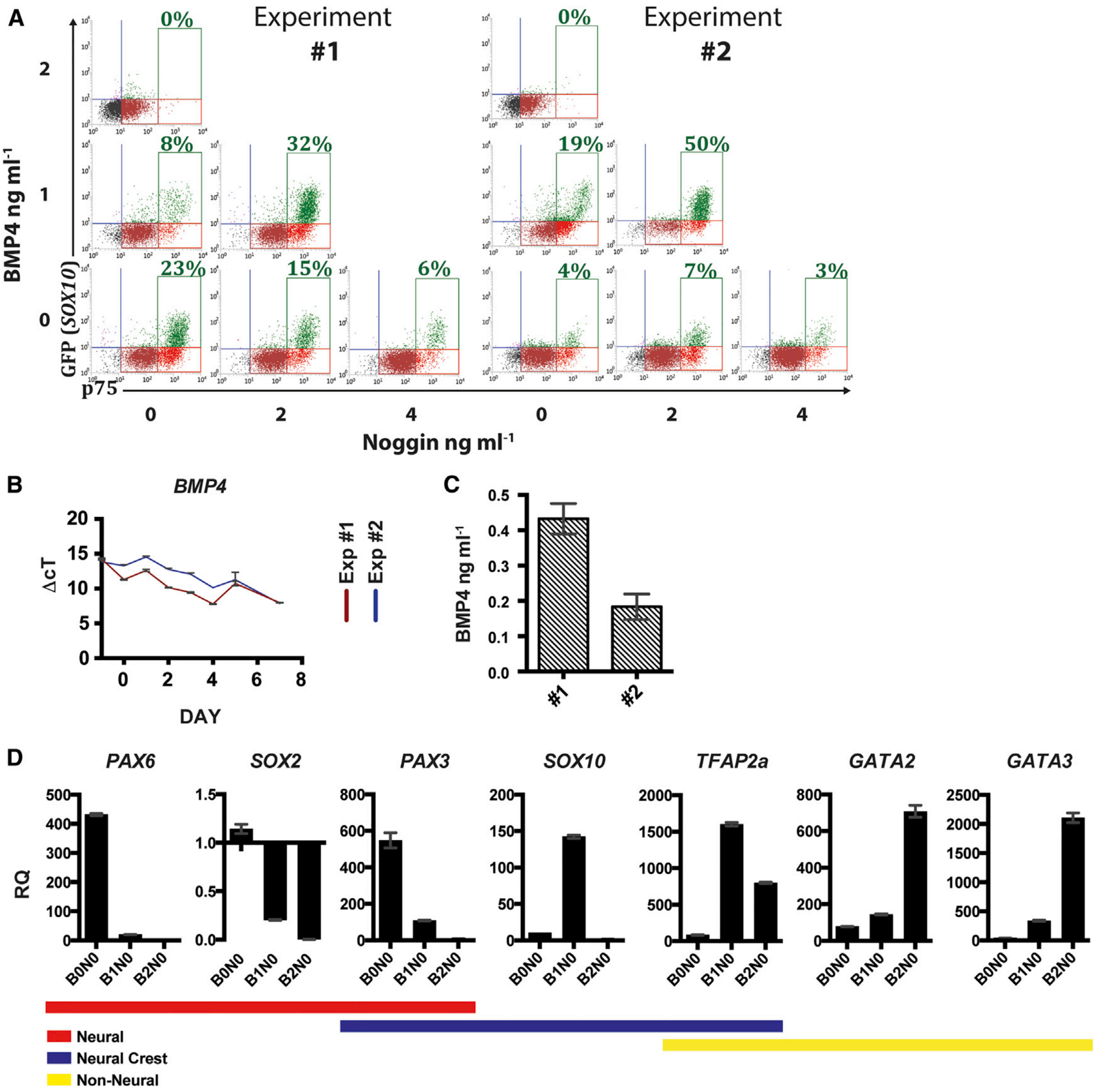

Figure 3. Endogenous BMP Expression Affects Neural Crest Differentiation Efficiency

(A) Day 7 flow cytometric analysis showing p75 and GFP expression in parallel cultures with varying concentrations of BMP4 and/or noggin (H9:*SOX10*). In the absence of these factors, differentiation efficiency ($p75^{++}$ GFP^{+}) was higher in experiment 1 than in experiment 2. 1 ng mL^{-1} BMP4 promoted neural crest in experiment 2 but repressed neural crest in experiment 1.

(B) qPCR shows significantly higher *BMP4* expression in experiment 1 than in experiment 2 between days 0 and 4 without exogenous BMP4 or noggin.

(C) BMP4 protein (ELISA) in the medium at day 0 was higher in experiment 1 without exogenous BMP4 or noggin.

(D) Day 7 gene expression (qPCR) in the presence of 0, 1, and 2 ng mL^{-1} exogenous BMP4 without noggin (experiment 2). A changing pattern consistent with a neural, neural crest, and non-neural identity, respectively, is observed. Colored bars denote tissue association. Error bars represent SD of technical replicates within corresponding experiment. All data shown represent neural crest induction using the cell line H9:*SOX10*.

Neural crest differentiation appears to require a precise level of BMP, which is confounded in different experiments due to variation in the endogenous production of BMP by hPSC cultures, perhaps caused by variable and uncontrolled spontaneous differentiation. Due to the difficulty in predicting the level of *BMP4* expression in hPSC cultures prior to neural crest differentiation, we instead designed a system to control the BMP signaling environment of the cultures irrespective of the level of endogenous BMP production. This system, termed "top-down inhibition" (TDi), involves

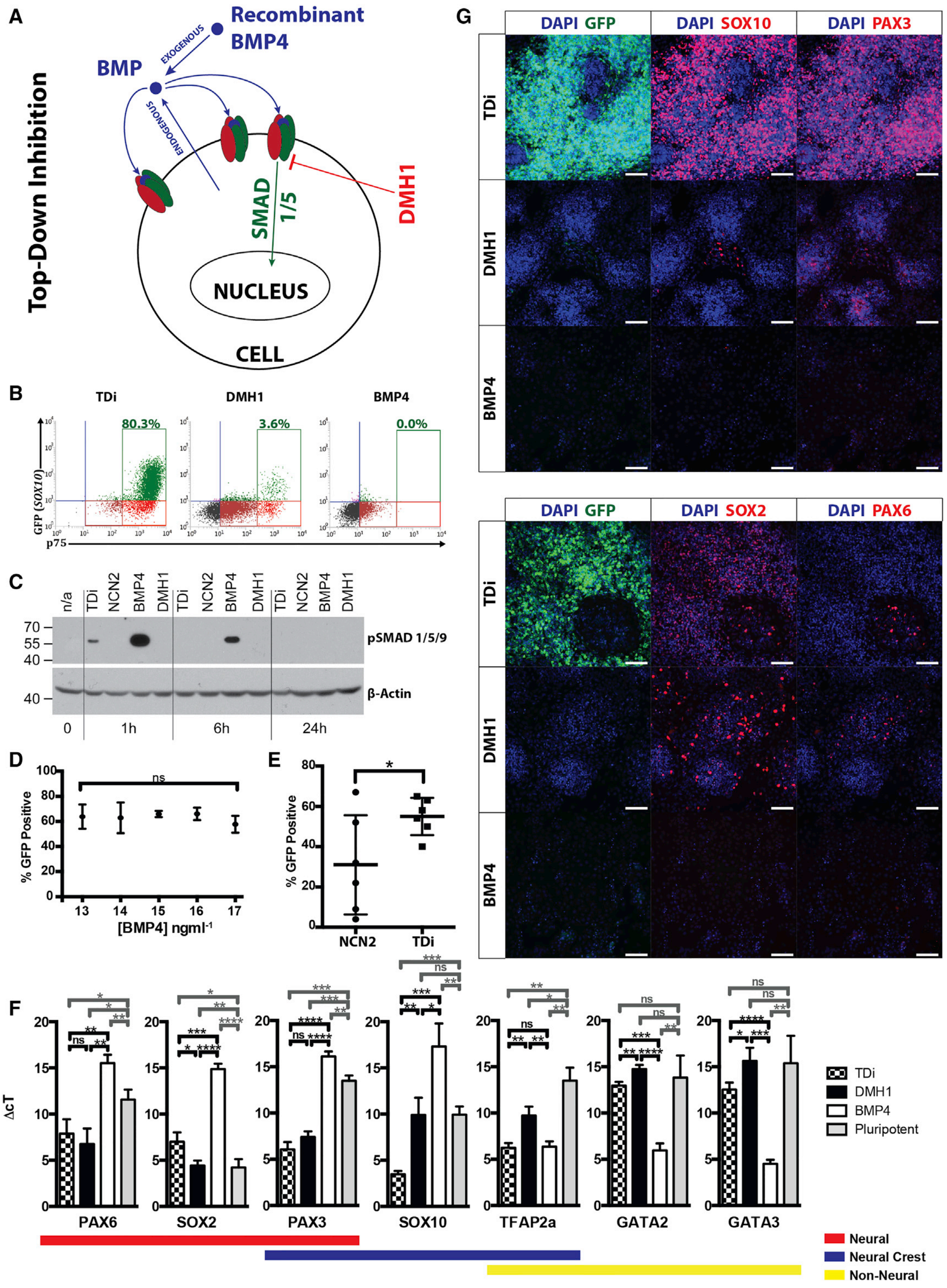

Figure 4. Top-Down Inhibition Controlling for the Effect of Variable Endogenous BMP Expression Using Agonist Saturation

(A) TDi schematic. Saturating with BMP4 means any variation in BMP levels occur outside of the cellular dose response; DMH1 attenuates the positive BMP signal.

(B) Flow cytometric analysis GFP expression in H9:*SOX10* at day 7 in TDi or medium containing either DMH1 or BMP4 alone.

(C) Presence of phospho-SMAD1/5/9 after switching to differentiation medium.

(legend continued on next page)

saturating cultures with recombinant BMP4 and simultaneously attenuating this signal using the BMP type 1 receptor inhibitor, DMH1 (Figure 4A). By adding high levels of recombinant BMP4, the range of BMP activity within which cells can respond is exceeded, and any change in endogenous BMP4 expression no longer results in a dose response from the culture. Application of DMH1 then allows the optimal BMP activity required for neural crest differentiation to be achieved without the induction of genes associated with non-neural ectoderm (Figures 4B, 4C, 4F, and 4G). A saturating amount of BMP4 (15 ng mL^{-1}) and an optimum amount of DMH1 (1.0 μM) were found by titration of these factors against one another. Use of DMH1 or BMP4 in the medium alone results in failure to induce *SOX10* expression (Figures 4B, 4F, and 4G). In the case of DMH1, the neural-associated transcription factors *PAX3* and *PAX6* are still expressed within the culture, as is *SOX2* (at a higher level in a smaller proportion of the cells). In the case of BMP4, *TFAP2a* is instead retained, and expression of the non-neural ectoderm-markers *GATA2* and *GATA3* is induced. Balancing both BMP4 and DMH1 creates an intermediate effect resulting in a neural plate border fate that is consistent across different hPSC lines (Figure S3).

Changing the level of BMP4 in TDi medium between 13 and 17 ng mL^{-1} did not significantly change the proportion of cells inducing *SOX10* expression (as reported by GFP), suggesting that 15 ng mL^{-1} is enough to saturate the system (Figure 4D). Western blot analysis shows that an hour after switching from seeding medium into TDi, levels of phospho-SMAD1/5/9 increased to a level that was less than in the presence of BMP4 alone but more than in the presence of DMH1 alone and NCN2 without treatment in this case (Figure 4C). These data also show that phospho-SMAD1/5/9 levels decrease with time despite the medium remaining unchanged. Most strikingly, with the use of TDi, we have been able to greatly reduce the variation exhibited between iterations of neural crest differentiation and thus improve the reproducibility of our experiments in all three cell lines tested (Figures 4E and S3). Differentiation of hPSCs into neural crest using TDi was also achieved on the xeno-free matrices Laminin 521 and vitronectin (Figure S4).

DISCUSSION

In this study, we developed a fully defined and xeno-free differentiation medium that can be used to generate putative neural crest from hPSCs in a robust manner. This medium can be used in conjunction with Laminin 521 or vitronectin as a substrate, allowing fully xeno-free production of neural crest for clinical applications and for further study into neural crest induction from hPSCs. Cells generated in this way can be maintained in non-adherent conditions in a manner that may allow for scaling up of neural crest cell production. The efficacy of our protocol is supported by the expression of neural crest-associated genes and surface markers in multiple cell lines, as well as functional analysis of differentiation capacity and motility. Although it is possible that a small proportion of the cells in culture at day 7 may be either mesenchymal or SOX10-positive cell types that are not neural crest, the majority of these cells exhibit marker expression (such as co-expression of SOX10 with PAX3 or PAX7) and function that is consistent with neural crest. Restriction of *SOX10* and *PAX6* expression to $p75^{++}$ and $p75^{+}$ populations, respectively, indicates that cells sorted on the basis of *SOX10* induction for peripheral neural differentiation are unlikely to contain neurectoderm precursors. The possibility that this system restricts further differentiation into the melanocyte lineage cannot be ruled out, as MITF positive cells could not be generated by following the protocol described by Mica et al. (2013), although melanocytes were not successfully generated from differentiation carried out in NCN2 either, so it is unlikely that this is caused by BMP saturation.

Intermediate levels of BMP signaling play a key inductive role in neural crest formation *in vivo*, but to date there has not been a consensus on the required level of BMP activity for neural crest differentiation of hPSCs *in vitro*. The results presented here, from work in a fully defined system, imply that intermediate levels of BMP activity are also required for human neural crest induction *in vitro*, and that levels outside of this narrow range lead to increased expression of transcription factors associated with other ectodermal tissues at the expense of those associated with neural crest. It is likely that in culture conditions that may be rich in

(D) Changes in the BMP4 concentration of TDi medium (15 ± 2 ng mL^{-1}) do not result in a significant change in neural crest efficiency.
(E) TDi significantly increases the reproducibility of differentiation. Presented is neural crest efficiency from the last six results using NCN2 and the first six results using TDi (H9:SOX10 within 6 months).
(F) Ectodermal gene expression at day 7 in TDi or medium containing DMH1 or BMP4 alone (qPCR). Association of transcription factors with ectodermal tissue is marked below. Data were pooled from three independent experiments.
(G) Expression of SOX10, PAX3, SOX2, PAX6, and GFP in H9:SOX10 at day 7 in TDi or in medium containing either DMH1 or BMP4 alone. Scale bars represent 100 μM. Error bars represent SD.
$*p \leq 0.05$, $**p \leq 0.01$, $***p \leq 0.001$, $****p \leq 0.0001$. ns, not significant. All data shown uses H9:*SOX10*. For supporting data using Shef6 (hESC) and Miff-1 (hiPSC), refer to Figure S3.

BMP, such as those containing KOSR, BMP inhibition would be required early during differentiation to prevent induction of a non-neural ectoderm fate. This reconciles the apparently conflicting published approaches to BMP signaling in neural crest differentiation from hPSCs. It is only in a fully defined system that the required signals for induction can be dissected, although endogenously produced growth factors can still be a confounding factor in such investigations. The inclusion of BSA may also effect the signaling environment during neural crest differentiation. BSA acts as a carrier protein facilitating uptake of some growth factors, and other ligands, by cells in culture (Francis, 2010). BSA purified from bovine serum may already be bound to biologically active factors that could affect differentiation or affect the activity of other factors added to the culture medium. Inclusion of BSA may also decrease the potency of the small molecules used for neural crest differentiation. Two other published systems for neural crest differentiation from hPSCs, both of which involve the use of BSA, require a 5-fold and 10-fold higher concentration of CHIR99021 and SB431542, respectively (Menendez et al., 2011; Leung et al., 2016).

In the development of this system, we showed that endogenous BMP expression can be sufficient to direct hPSCs toward an neural crest fate in an environment that is devoid of external BMP modulation (using NCN2 medium). However, we encountered difficulties generating neural crest with reproducible differentiation efficiencies while using this basic system. By using TDi, we were able to significantly reduce this variability, presumably by stabilizing the level of BMP activity between experiments. It should be noted that recombinant BMP4 from different sources may vary in activity and should therefore be titrated in the presence of 1 μM DMH1 to assess the saturation point on the basis of efficient neural crest induction. The TDi approach also has the advantage that it controls for the effect of endogenously produced BMP accumulation in the medium during periods between media changing. A caveat to this approach is that flooding the system with recombinant BMP4 will change the ratio of BMP4 to other BMP ligands, although this does not appear to adversely affect neural crest induction. The observation that phospho-SMAD1/5/9 peaks 1 hr after application of differentiation medium, before decreasing, despite the medium remaining unchanged, is consistent with observations of phospho-SMAD4 induction and turnover in response to TGF-b signaling (Sorre et al., 2014).

TDi could be used in any system where an attenuated signal is required and an agonist and antagonist are available that act extracellularly and intracellularly, respectively, with a high level of specificity. The small molecule DMH1 is ideal for this as it acts on the BMP type 1 receptor (Hao et al., 2010), as opposed to antagonists such as noggin that bind BMP ligands directly (Groppe et al., 2002). If such molecules are not available, an alternative "baseline activation" approach would be to saturate instead with an antagonist that binds directly to any endogenously produced ligand in the medium, used in conjunction with a small molecule that activates the signaling pathway internally (examples for the WNT pathway could be DKK1 and CHIR99021, respectively). Approaches such as these would be particularly useful when recapitulating the concentration-dependent effects of morphogens or when endogenous signal expression is a confounding factor. Although using TDi greatly reduced the variability observed between iterations of neural crest differentiation and showed robust differentiation in all lines tested, some variability still exists, raising the possibility that endogenous expression of other signals, such as WNT, may also be having an effect. Like BMP, titration of WNT3a or CHIR99021 shows an optimum level of WNT activity for neural crest induction (data not shown). This raises the possibility that endogenous production of WNT ligands may also adversely affect differentiation. Finally, it is to be noted that heterogeneity still persists in neural crest differentiation cultures and that although some of the cells that are SOX10 negative on day 7 may still be in a precursor stage, occasional rosette-like structures and other morphological characteristics within the culture suggest that others have more in common with neural or possibly non-neural ectoderm (Figures 4G and S3B). This may be due to different signaling microenvironments within the culture or cells within the original hPSC population that exhibit bias toward particular fates.

Conclusion

Here, we present a fully defined and xeno-free system for neural crest induction from hPSCs. We identify endogenous BMP production as a major source of variability in neural crest differentiation efficiency and describe a method that controls this variability, the theory of which could be applied in other protocols for the differentiation of hPSCs.

EXPERIMENTAL PROCEDURES

Pluripotent Stem Cell Culture

All cell lines were cultured in mTESR on Matrigel. See Supplemental Experimental Procedures for more details.

Neural Crest Differentiation

Neural crest differentiation was achieved as described in the Results.

Non-adherent Culture of Neural Crest

Day 7 cultures were detached using Accutase (Gibco, A11105-01; 7 min); 50,000–100,000 cells were added per well in 96-well plates

coated with 1% agarose in N2 supplemented DMEM F12 containing ROCK inhibitor (γ-27632) for the first 24 hr and with half media changes every 2–3 days.

Immunofluorescence and Flow Cytofluorimetry

For details including a list of antibodies, see Supplemental Experimental Procedures.

qPCR

The Taqman gene expression assay system was used for all qPCR. For details including a list of assay mixes, see Supplemental Experimental Procedures.

Terminal Differentiation of Neural Crest

Terminal differentiation into mesectodermal and neural lineages was performed using kits from STEMCELL Technologies and protocols published by the Studer lab (Lee et al., 2010; Chambers et al., 2012), respectively. For details, see Supplemental Experimental Procedures.

SUPPLEMENTAL INFORMATION

Supplemental Information includes Supplemental Experimental Procedures and four figures and can be found with this article online at http://dx.doi.org/10.1016/j.stemcr.2017.08.008.

AUTHOR CONTRIBUTIONS

J.O.S.H. and C.U. carried out the initial protocol design. TDi was conceived by J.O.S.H., and the project was directed by P.W.A. J.O.S.H. designed and carried out the experiments and wrote the manuscript. T.J.R.F., J.O.S.H., A.M.N., and C.U. carried out functional analysis by differentiation. M.I.G.C. provided critical expertise, support and resources while addressing reviewers comments. O.T. and A.M.N. performed western blots and laminin screens for neural crest differentiation, respectively.

ACKNOWLEDGMENTS

We thank Lorenz Studer, Mark Tomishima, and Yvonne Mica for kindly providing the H9: *SOX10* BAC hESC line to us, Mark Jones for FACS assistance, Paul Gokhale for advice, and Anestis Tsakaridis and Ivana Barbaric for critically reviewing the manuscript. This work was supported in part by grants from the Medical Research Council (R/143416-14-1), the European Community's Seventh Framework Programme (FP7/2007-2013 under grant agreement no. 602423), Yorkshire Cancer Research (S002 PhD), and Barncancerfonden.

Received: January 23, 2017
Revised: August 14, 2017
Accepted: August 14, 2017
Published: September 14, 2017

REFERENCES

Barth, K.A., Kishimoto, Y., Rohr, K.B., Seydler, C., Schulte-Merker, S., and Wilson, S.W. (1999). Bmp activity establishes a gradient of positional information throughout the entire neural plate. Development *126*, 4977–4987.

Basch, M.L., Bronner-Fraser, M., and García-Castro, M.I. (2006). Specification of the neural crest occurs during gastrulation and requires Pax7. Nature *441*, 218–222.

Betters, E., Liu, Y., Kjaeldgaard, A., Sundström, E., and García-Castro, M.I. (2010). Analysis of early human neural crest development. Dev. Biol. *344*, 578–592.

Chambers, S.M., Fasano, C.A., Papapetrou, E.P., Tomishima, M., Sadelain, M., and Studer, L. (2009). Highly efficient neural conversion of human ES and iPS cells by dual inhibition of SMAD signaling. Nat. Biotechnol. *27*, 275–280.

Chambers, S.M., Qi, Y., Mica, Y., Lee, G., Zhang, X.J., Niu, L., Bilsland, J., Cao, L., Stevens, E., Whiting, P., et al. (2012). Combined small-molecule inhibition accelerates developmental timing and converts human pluripotent stem cells into nociceptors. Nat. Biotechnol. *30*, 715–720.

Chen, W., Jongkamonwiwat, N., Abbas, L., Eshtan, S.J., Johnson, S.L., Kuhn, S., Milo, M., Thurlow, J.K., Andrews, P.W., Marcotti, W., et al. (2012). Restoration of auditory evoked responses by human ES-cell-derived otic progenitors. Nature *490*, 278–282.

Fattahi, F., Steinbeck, J.A., Kriks, S., Tchieu, J., Zimmer, B., Kishinevsky, S., Zeltner, N., Mica, Y., El-Nachef, W., Zhao, H., et al. (2016). Deriving human ENS lineages for cell therapy and drug discovery in Hirschsprung disease. Nature *531*, 105–109.

Faure, S., Lee, M.A., Keller, T., ten Dijke, P., and Whitman, M. (2000). Endogenous patterns of TGFbeta superfamily signaling during early *Xenopus* development. Development *127*, 2917–2931.

Faure, S., de Santa Barbara, P., Roberts, D.J., and Whitman, M. (2002). Endogenous patterns of BMP signaling during early chick development. Dev. Biol. *244*, 44–65.

Francis, G.L. (2010). Albumin and mammalian cell culture: implications for biotechnology applications. Cytotechnology *62*, 1–16.

Furthauer, M., Thisse, B., and Thisse, C. (1999). Three different noggin genes antagonize the activity of bone morphogenetic proteins in the zebrafish embryo. Dev. Biol. *214*, 181–196.

Groppe, J., Greenwald, J., Wiater, E., Rodriguez-Leon, J., Economides, A.N., Kwiatkowski, W., Affolter, M., Vale, W.W., Izpisua Belmonte, J.C., and Choe, S. (2002). Structural basis of BMP signalling inhibition by the cystine knot protein Noggin. Nature *420*, 636–642.

Hao, J., Ho, J.N., Lewis, J.A., Karim, K.A., Daniels, R.N., Gentry, P.R., Hopkins, C.R., Lindsley, C.W., and Hong, C.C. (2010). In vivo structure-activity relationship study of dorsomorphin analogues identifies selective VEGF and BMP inhibitors. ACS Chem. Biol. *5*, 245–253.

Hemmati-Brivanlou, A., Kelly, O.G., and Melton, D.A. (1994). Follistatin, an antagonist of activin, is expressed in the Spemann organizer and displays direct neuralizing activity. Cell *77*, 283–295.

LaBonne, C., and Bronner-Fraser, M. (1998). Neural crest induction in *Xenopus*: evidence for a two-signal model. Development *125*, 2403–2414.

Lamb, T.M., Knecht, A.K., Smith, W.C., Stachel, S.E., Economides, A.N., Stahl, N., Yancopolous, G.D., and Harland, R.M. (1993).

Neural induction by the secreted polypeptide noggin. Science *262*, 713–718.

Lee, G., Chambers, S.M., Tomishima, M.J., and Studer, L. (2010). Derivation of neural crest cells from human pluripotent stem cells. Nat. Protoc. *5*, 688–701.

Leung, A.W., Murdoch, B., Salem, A.F., Prasad, M.S., Gomez, G.A., and García-Castro, M.I. (2016). WNT/beta-catenin signaling mediates human neural crest induction via a pre-neural border intermediate. Development *143*, 398–410.

Liu, Q., Spusta, S.C., Mi, R., Lassiter, R.N., Stark, M.R., Höke, A., Rao, M.S., and Zeng, X. (2012). Human Neural crest stem cells derived from human ESCs and induced pluripotent stem cells: induction, maintenance, and differentiation into functional Schwann cells. Stem Cells Transl. Med. *1*, 266–278.

Marchant, L., Linker, C., Ruiz, P., Guerrero, N., and Mayor, R. (1998). The inductive properties of mesoderm suggest that the neural crest cells are specified by a BMP gradient. Dev. Biol. *198*, 319–329.

Menendez, L., Yatskievych, T.A., Antin, P.B., and Dalton, S. (2011). Wnt signaling and a Smad pathway blockade direct the differentiation of human pluripotent stem cells to multipotent neural crest cells. Proc. Natl. Acad. Sci. USA *108*, 19240–19245.

Metallo, C.M., Ji, L., de Pablo, J.J., and Palecek, S.P. (2010). Directed differentiation of human embryonic stem cells to epidermal progenitors. Methods Mol. Biol. *585*, 83–92.

Mica, Y., Lee, G., Chambers, S.M., Tomishima, M.J., and Studer, L. (2013). Modeling neural crest induction, melanocyte specification, and disease-related pigmentation defects in hESCs and patient-specific iPSCs. Cell Rep. *3*, 1140–1152.

Nguyen, V.H., Schmid, B., Trout, J., Connors, S.A., Ekker, M., and Mullins, M.C. (1998). Ventral and lateral regions of the zebrafish gastrula, including the neural crest progenitors, are established by a bmp2b/swirl pathway of genes. Dev. Biol. *199*, 93–110.

O'Rahilly, R., and Müller, F. (2007). The development of the neural crest in the human. J. Anat. *211*, 335–351.

Pera, M.F., Andrade, J., Houssami, S., Reubinoff, B., Trounson, A., Stanley, E.G., Ward-van Oostwaard, D., and Mummery, C. (2004). Regulation of human embryonic stem cell differentiation by BMP-2 and its antagonist noggin. J. Cell Sci. *117*, 1269–1280.

Piccolo, S., Sasai, Y., Lu, B., and De Robertis, E.M. (1996). Dorsoventral patterning in *Xenopus*: inhibition of ventral signals by direct binding of chordin to BMP-4. Cell *86*, 589–598.

Sasai, Y., Lu, B., Piccolo, S., and De Robertis, E.M. (1996). Endoderm induction by the organizer-secreted factors chordin and noggin in *Xenopus* animal caps. EMBO J. *15*, 4547–4555.

Selleck, M.A., and Bronner-Fraser, M. (1995). Origins of the avian neural crest: the role of neural plate-epidermal interactions. Development *121*, 525–538.

Sorre, B., Warmflash, A., Brivanlou, A.H., and Siggia, E.D. (2014). Encoding of temporal signals by the TGF-beta pathway and implications for embryonic patterning. Dev. Cell *30*, 334–342.

Steventon, B., Araya, C., Linker, C., Kuriyama, S., and Mayor, R. (2009). Differential requirements of BMP and Wnt signalling during gastrulation and neurulation define two steps in neural crest induction. Development *136*, 771–779.

Timmer, J.R., Wang, C., and Niswander, L. (2002). BMP signaling patterns the dorsal and intermediate neural tube via regulation of homeobox and helix-loop-helix transcription factors. Development *129*, 2459–2472.

Tribulo, C., Aybar, M.J., Nguyen, V.H., Mullins, M.C., and Mayor, R. (2003). Regulation of Msx genes by a Bmp gradient is essential for neural crest specification. Development *130*, 6441–6452.

Wilson, P.A., Lagna, G., Suzuki, A., and Hemmati-Brivanlou, A. (1997). Concentration-dependent patterning of the *Xenopus* ectoderm by BMP4 and its signal transducer Smad1. Development *124*, 3177–3184.

Workman, M.J., Mahe, M.M., Trisno, S., Poling, H.M., Watson, C.L., Sundaram, N., Chang, C.F., Schiesser, J., Aubert, P., Stanley, E.G., et al. (2016). Engineered human pluripotent-stem-cell-derived intestinal tissues with a functional enteric nervous system. Nat. Med. *23*, 49–59.

Zimmerman, L.B., De Jesus-Escobar, J.M., and Harland, R.M. (1996). The Spemann organizer signal noggin binds and inactivates bone morphogenetic protein 4. Cell *86*, 599–606.

Stem Cell Reports, Volume *9*

Supplemental Information

Top-Down Inhibition of BMP Signaling Enables Robust Induction of hPSCs Into Neural Crest in Fully Defined, Xeno-free Conditions

James O.S. Hackland, Tom J.R. Frith, Oliver Thompson, Ana Marin Navarro, Martin I. Garcia-Castro, Christian Unger, and Peter W. Andrews

Supplemental Data

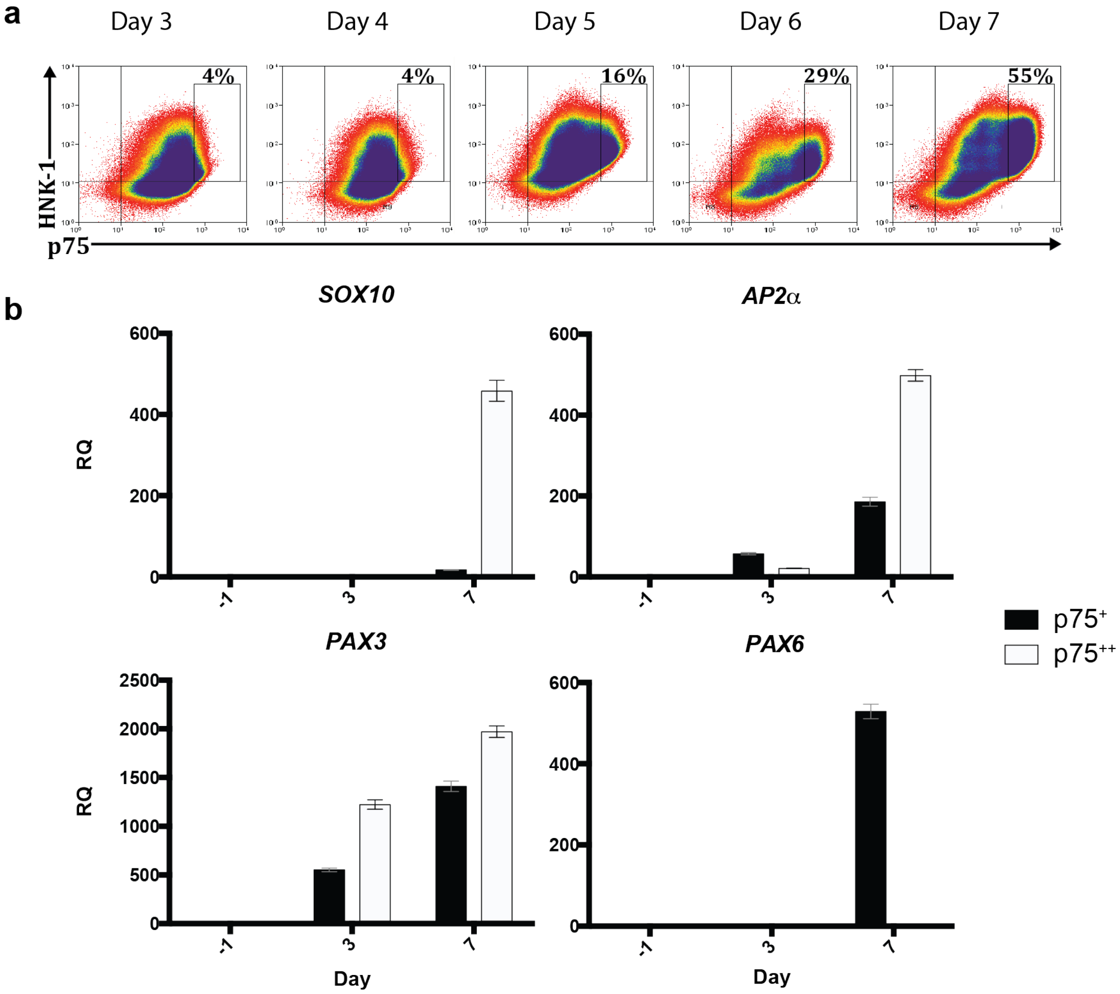

Figure S1. Gene expression in p75 high/low ($p75^{++}/p75^{+}$) during neural crest induction of Shef6 using NCN2. Related to Figure 1. (**a**) The surface marker p75 is upregulated during neural crest induction and (**b**) can be used to purify the culture for *SOX10* expressing cells in the absence of a *SOX10* reporter construct. Data represents a single experiment showing typical distribution of gene expression between populations.

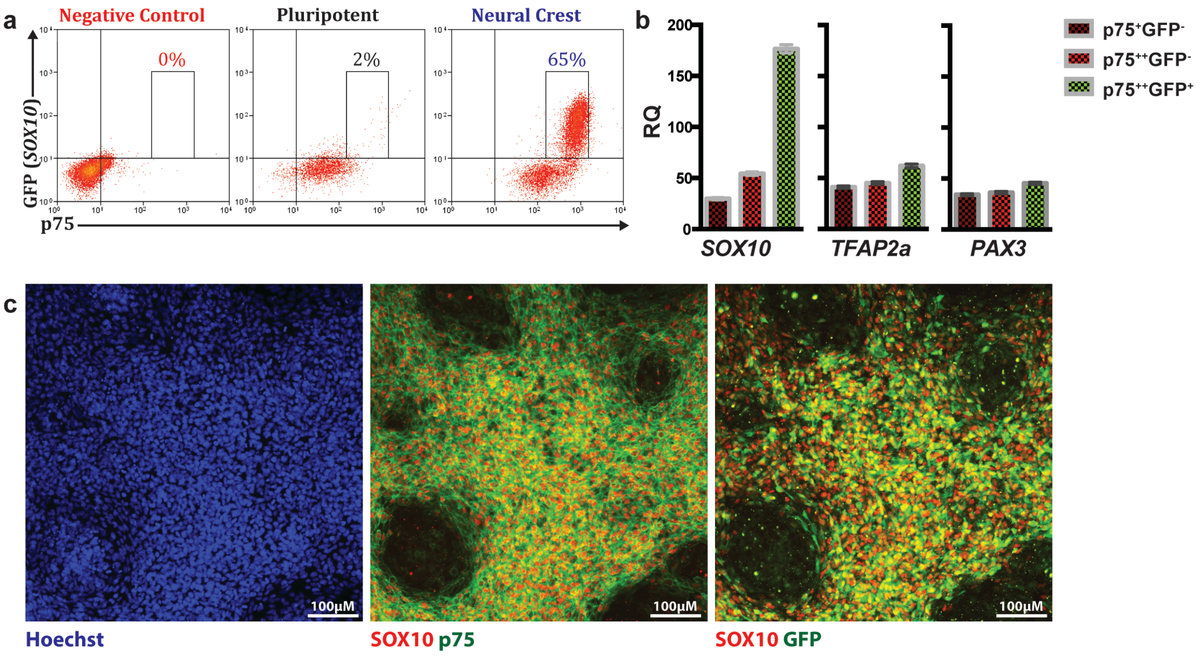

Figure S2. Expression of surface markers and neural crest-associated genes in H9:*SOX10*. Related to Figures 2, 3 & 4. (a) Expression of GFP and p75 in pluripotent cells and after 7 days of neural crest differentiation. (b) Expression of neural crest-associated genes after FACS in p75+, p75++ and p75++GFP+ populations after neural crest differentiation. Data represents a single experiment showing technical replicates. (c) Expression of SOX10, p75 and GFP in a typical culture at day 7 of neural crest differentiation.

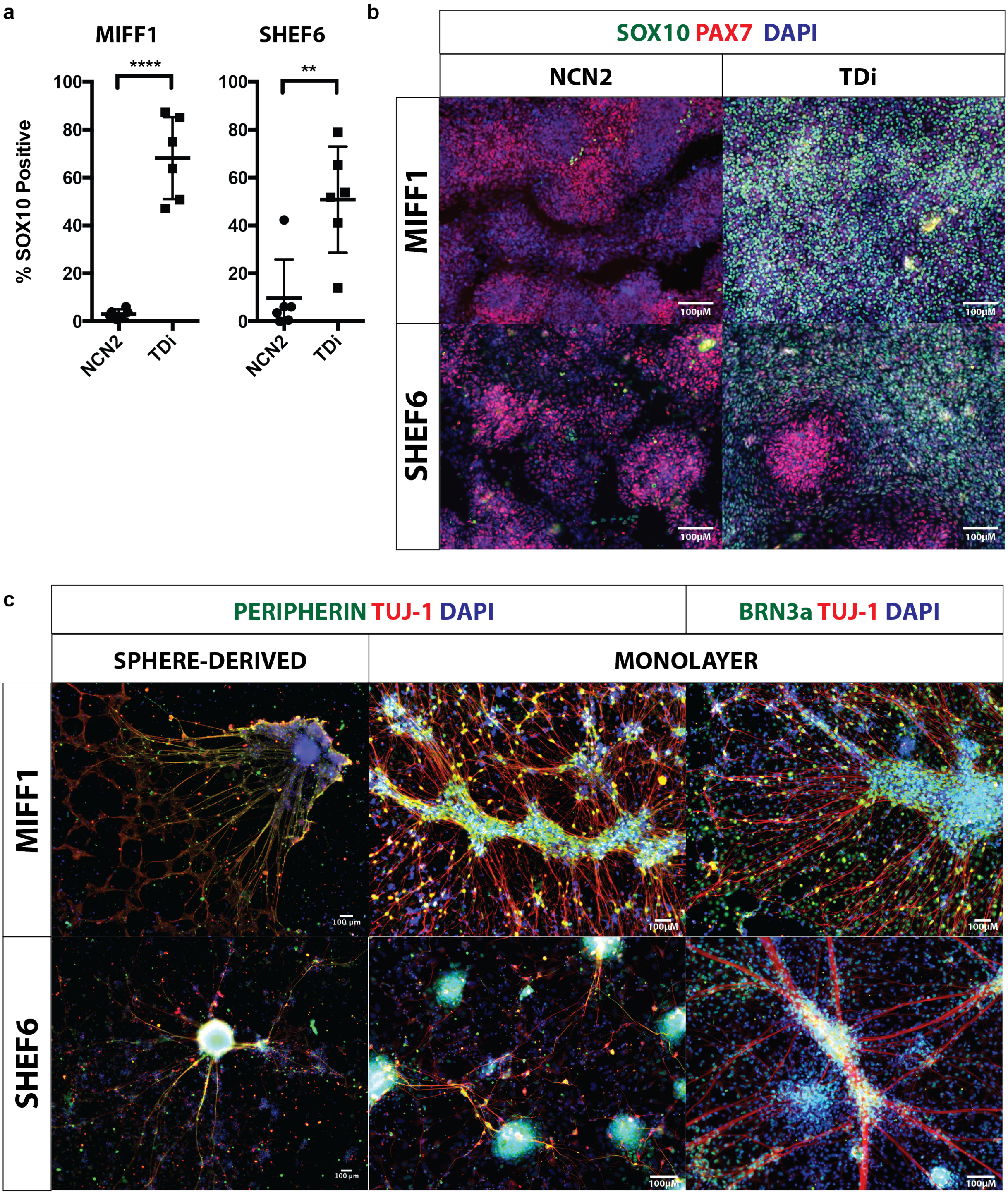

Figure S3. Neural crest induction using TDi in Shef6 (hESC) and Miff-1 (hiPSC). Related to Figure 4. (**a**) Use of TDi during neural crest induction significantly improves the reproducibility of differentiation in both Shef6 and Miff-1. Each point represents a single experiment. Error bars represent SD. *P≤0.05, **P≤0.01, ***P≤0.001, ****P≤0.0001. Efficiency of induction was calculated by applying Nikon Elements image analysis software to immunofluorescence data examples of which are shown in (**b**). (**b**) Typical expression of neural crest markers SOX10 and PAX7 after 7 days of induction using either NCN2 or TDi. (**c**) Neural crest generated from Shef6 and Miff-1 respond to neural induction conditions, either in monolayer or from sphere culture, by expressing the peripheral neural marker Peripherin and the sensory neural marker Brn3a.

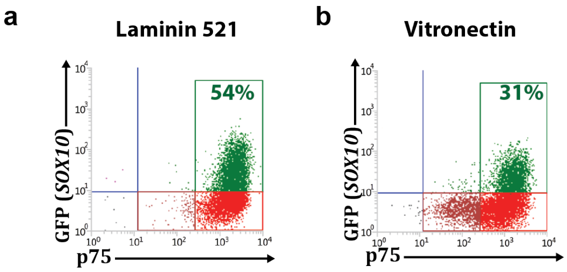

Figure S4. Neural Crest differentiation on other matrices. Related to differentiation in fully defined and xeno-free conditions. Neural crest differentiation of H9:*SOX10* on fully defined and xeno-free matrices Laminin-521 and vitronectin using TDi.

**Antibody**	**Supplier/Catalog Number**
**Ms anti PAX3**	**DSHB/AB528426**
**Ms anti PAX6**	**Santa Cruz/sc81649**
**Rb anti SOX10**	**Cell signaling/89356S**
**Rb anti SOX2**	**Cell signaling/3579P**
**Rb anti p75**	**Cell signaling/8238**
**Ms anti HNK-1**	**Sigma/C6680**
**Chk anti GFP**	**Abcam/ab13970**
**Ms anti S100b**	**Sigma/S2532**

List of antibodies used for FACS and immunofluorescence.

**Gene**	**Assay ID**
SOX10	**Hs00366918_m1**
TFAP2a	**Hs01029413_m1**
PAX3	**Hs00240950_m1**
PAX6	**Hs01088114_m1**
SOX2	**Hs01053049_s1**
GATA2	**Hs00231119_m1**
GATA3	**Hs00231122_m1**
OCT4 (POU5F1)	**Hs04260367_gH**

List of Taqman Gene Expression Assay mixes used (Catalog Number 4331182).

Supplemental Experimental Procedures

Pluripotent Stem Cell Culture

WA09 (H9) (Thomson et al. 1998), Shef6 (Aflatoonian et al. 2010), and H9:*SOX10* (Chambers et al. 2012) hESC, and Miff-1 (Desmarais et al. 2016), NB1 (unpublished, fully characterized) hiPSC, were cultured in mTESR on matrigel. All cells used had a normal diploid karyotype.

Neural Crest Differentiation

N2 supplement was sourced from ThermoFisher (17502048), CHIR99021, SB431542 and DMH1 were sourced from Tocris (4425, 1614, 4126 respectively) and BMP4 was from Life Technologies (PHC9534).

Immunofluorescence

Cultures were fixed in 4% paraformaldehyde for 10 minutes before permeabilization and blocking for 1 hour (1% BSA, 10% goat serum, 0.3M glycine and 0.1% Tween-20 in PBS). Primary and secondary antibody incubation was carried out in the same buffer overnight and for 1 hour respectively (see supplemental table). Hoechst 33342 (H3570 - Life Technologies) was used as a DNA stain. Images were taken using an InCell Analyser 2200 (GE Healthcare Life Sciences). For the data presented in Supplemental Figure 3 images were taken using a Nikon Eclipse Ti and analyzed using Nikon Elements image analysis software. These data were calculated as follows: Each biological replicate shown represents the mean expression values of three technical replicates each of which represent four preset locations within each well. The positive/negative intensity threshold was set in each case with the aid of an additional well without primary antibody.

Flow Cytofluorimetry

Flow cytometry analysis was carried out as previously described (Draper et al. 2002). Briefly, cultures were treated with Accutase (Gibco - A11105-01) and both primary and secondary antibody incubation was carried out in in FACS buffer (10% FCS, PBS) for 15 minutes (see supplemental table). Cells were analyzed using a BD FACSJazz (BD Biosciences). For negative controls the antibody produced by the parent myeloma P3X63AG8 was used (Kohler & Milstein 1975; Draper et al. 2002).

qPCR

RNA was extracted from whole cultures or sorted populations using TRIzol (15596018 - Life Technologies) and cDNA was generated using Multiscribe reverse transcriptase (4311235 - Life Technologies). qPCR was carried out using 4ng

of cDNA per 10ml reaction with Taqman Fast Universal PCR master mix (1411408 - Life Technologies) and pre-optimized Gene Expression Assay mixes (4331182 - Life Technologies; supplementary table) before analysis using the QuantStudio 12K Flex Real-Time PCR system (4471087 - Life Technologies). Data are presented either as difference in cycle threshold between the gene of interest (GOI) and β-Actin (ΔcT) or a relative quantification (RQ) of the change in gene expression between the initial pluripotent culture and the differentiated culture (RQ; 2^-ΔΔcT).

Terminal Differentiation of Neural Crest

Terminal differentiation was performed on GFP^+ sorted putative neural crest derived from H9:*SOX10* unless otherwise stated. For mesectoderm differentiation, cells were plated out in MesenCult™-ACF (StemCell Technologies Cat# 05449). After 10 days, medium was changed to MesenCult™-ACF Chondrogenic Differentiation Medium (StemCell Technologies Cat#05455) or Osteogenic Stimulatory Kit (StemCell Technologies Cat# 05404) and analyzed using Toluidine Blue (Acris Bioscience) Alizarin Red staining (Sigma A5533).

Terminal differentiation into neural lineages was carried out as described in Lee (2010) either as a monolayer or as neural crest spheres directly from non-adherent culture. Plates were coated with Fibronectin (Sigma F1141), Laminin (Cultrex 3400-010-01) and Poly-L-Ornithine (Sigma P4957). FGF, EGF, GDNF, BDNF and NGF were sourced from peprotech and dbCAMP from Sigma (D0627). The BRN3a and ISL1 expression shown in Figure 2d and Supplemental Figure 3 was achieved using the modified version of this protocol described in (Chambers et al. 2012).
